# Physical Health Among Black Immigrants by Region of Birth: A Test of the Racial Context Hypothesis

**DOI:** 10.1007/s40615-024-02167-x

**Published:** 2024-10-03

**Authors:** Oluwaseun T. Emoruwa, Gabe H. Miller, Gbenga I. Elufisan, Guadalupe Marquez-Velarde, David Ademule, Hannah M. Lindl, Olusola A. Omisakin, Guizhen Ma, Stephanie M. Hernandez, Verna M. Keith

**Affiliations:** 1 Department of Sociology, University of Alabama at Birmingham, 1720 2Nd Ave South, Birmingham, AL 32594-0110 USA; 2 Department of Sociology, Mississippi State University, Mississippi State, MS USA; 3 Department of Sociology and Anthropology, Utah State University, Logan, UT USA; 4 Population Research Institute, Pennsylvania State University, University Park, PA USA; 5 Division of Social Sciences and History, Delta State University, Cleveland, MS USA; 6 Department of Epidemiology and Biostatistics, Drexel University, Philadelphia, PA USA

**Keywords:** Black immigrants, Immigrant health, Racial context, Self-rated health, Activity limitation, Hypertension, Cancer

## Abstract

**Objective:**

We test the Racial Context Hypothesis by examining the association between racial context of origin and five physical health outcomes (self-rated health, activity limitation, functional activity limitation, lifetime hypertension, and lifetime cancer) among U.S.-born Black Americans and Black immigrants in the United States.

**Design:**

This cross-sectional study used 2000 through 2018 waves of the National Health Interview Survey (NHIS). Our subsample was limited to adults 18 years of age or older who self-identified as Black and selected a distinct global region of birth if not U.S. born (*N* = 212,269). We employed zero-order logistic regression models to estimate the relationships between each measure of health and racial context by region of birth.

**Results:**

Supporting the Racial Context Hypothesis, we found Black immigrants from racially mixed (Mexico, Central America, the Caribbean, South America) and majority-Black contexts (Africa) had lower odds of being in fair or poor self-rated health [aOR 0.786; 0.616; 0.611], reporting any activity limitation [aOR = 0.537; 0.369; 0.678], reporting functional activity limitation [aOR 0.619; 0.425; 0.678], reporting lifetime hypertension diagnosis [aOR 0.596; 0.543; 0.618], and reporting lifetime cancer diagnosis [aOR 0.771; 0.326; 0.641] compared to U.S.-born Black Americans. After controlling for sociodemographic and socioeconomic covariates, Black immigrants from majority-White contexts (Europe) did not significantly differ from U.S.-born Black Americans on these five physical health measures.

**Conclusion:**

This study expands our understanding of the “Black immigrant advantage” by showing that Black immigrants from predominantly Black and racially mixed regions rated their health status as poor or fair less often, experienced less activity or functional activity limitations, and had a lower risk of lifetime hypertension and cancer compared to U.S.-born Black Americans. The significant associations persisted even after controlling for sociodemographic and socioeconomic characteristics. Black immigrant health is not homogenous, and the racial context of origin Black immigrants come from has an association with their health outcomes.

## Introduction

In response to the changes in the composition of the United States (U.S.) population due to global migration streams of diverse provenance, researchers are increasingly examining the health of those groups of immigrants in comparison with that of native-born individuals in order to better understand the health trends of the U.S. population. Black immigrants in the U.S. currently make up one-tenth of the Black population in the country, with predictions that this proportion will rise to 16.5% by 2060 [1]. The growth of the Black immigrant population has profound implications for the overall Black population in the U.S. Black immigrants have different health patterns across health outcomes when compared to U.S.-born Black individuals [2]. U.S.-born Black individuals report worse health status and outcomes compared to other racial and ethnic groups, and racial discrimination is one of the most significant causes of social inequities in health [3, 4]. While many studies have examined the effects of racial discrimination on the health of U.S.-born Black individuals, recent work has begun to consider the mechanisms through which racial discrimination influences the health of Black immigrants [5–9].

This study extends previous research by examining five health outcomes (self-rated health, activity limitation, functional activity limitation, lifetime hypertension, and lifetime cancer) among Black immigrants from various regions of origin (1) Africa, (2) South America, (3) Mexico, Central America, and the Caribbean, and (4) Europe) compared to U.S.-born Black Americans using pooled data from the 2000–2018 National Health Interview Survey (NHIS). Compared to U.S.-born Black Americans, Black immigrants to the U.S. tend to have longer life expectancy [10] and lower morbidity and mortality rates [11]. Black immigrants are more likely to report their health as “excellent,” have lower family cancer prevalence [12], report fewer chronic medical conditions and have lower rates of activity limitation [13], have lower rates of HIV infection [14], lower odds of stroke and stroke mortality [15], and are less likely to suffer from hypertension [16]. Nevertheless, prolonged residence in the U.S. tends to erode this health advantage [8, 17].

In this study, our primary research objective is to examine variation in self-rated health, activity limitation, functional activity limitation, lifetime hypertension, and lifetime cancer by racial context of origin. Thus, we test the racial context of origin hypothesis [5] against five health outcomes. Specifically, we extend Read & Emerson’s original analysis by including additional health outcomes as well as expanding the initial data used to include data from 2000 to 2018. Building upon the racial context of origin hypothesis, we expect that Black immigrants from predominantly Black or racially mixed regions of origin will report better health compared to Black immigrants from majority-White contexts and U.S.-born Black Americans. Furthermore, we anticipate that Black immigrants from predominantly White contexts will have similar health patterns to U.S.-born Black individuals as a result of greater exposure to racial discrimination.

## The Black Immigrant Health Advantage

There is consistent evidence that Black immigrants in the U.S. and U.S.-born Black individuals have different health patterns. Black immigrants in the U.S. report similar outcomes as U.S.-born White Americans, and therefore hold a health advantage across a variety of health outcomes [2, 5–7, 18]. For instance, compared to their U.S.-born counterparts, Black immigrants are less likely to suffer from hypertension [18], have lower rates of functional limitation [19], lower risk of low infant birth weight [20], lower prevalence of heart-related diseases [21], and are less likely to report poor or fair health status [8, 22]. There are, however, different patterns for other diseases, where Black immigrants are less healthy than U.S.-born Black Americans. For example, Black immigrants from Africa have a higher tuberculosis rate when compared to US-born Black and White adults [23], and although Black immigrants have an overall lower cancer mortality rate [12, 24, 25], Black immigrants from sub-Saharan Africa have more cases of infection-related cancers and blood cancers [26].

In general, Black immigrants tend to have health behaviors that are positively associated with health. For instance, compared to their U.S.-born counterparts, Black immigrants are less likely to be heavy drinkers [7], less likely to report being a current smoker [7, 27], have better dietary habits [12], and are less likely to be obese [28]. However, following a prolonged stay in the U.S., Black immigrants exhibit similar health status and behaviors to U.S.-born Black Americans [8, 17].

Although most studies on Black immigrants’ health focus on comparing Black immigrants to U.S.-born Black Americans across several health outcomes and socioeconomic status, an emerging body of literature has begun to examine heterogeneity among Black immigrants by considering specific country of origin. In addition to differences in racial composition in region of origin (Read and Emerson 2005), diversity exists on several social and economic factors [29] with implications for health outcomes in the U.S.. For instance, although Black immigrants from countries with higher life expectancy at birth, lower levels of income inequality, and higher education enrollment for school-aged children, had more favorable health status, after adjusting for country of birth characteristics, Black immigrants from Africa interestingly reported the best health despite having worse birth-country characteristics [11]. Consequently, Black immigrants from Africa reported the best health rating while Black immigrants from Central America reported the worst self-rated health status [11]. Even with recorded disadvantages on other socio-economic factors, Black immigrants from Nigeria have the highest average years of schooling while Black immigrants from Central America especially Mexico have the lowest mean years of education [8, 11]. Black immigrants also vary in family size and educational attainment. For example, Black immigrants from Caribbean regions typically have a smaller family size when compared to Black families from Africa and although Black immigrants from Africa have higher educational attainment than Black Caribbean immigrants, they have a wider gender gap among couples in educational attainment compared to Black Caribbean immigrant couples [30]. Using findings from three physical health outcomes: self-rated health, activity limitation, and hypertension, Read, Emerson, & Tarlov [9] disaggregated the Black immigrant population into three groups consisting of Black immigrants from Africa, West Indies, or Europe and compared them to U.S.-born Black and White adults. Consequently, Black immigrants from Africa were largely responsible for the Black immigrant advantage found in previous studies as they had better status on the three health outcomes, followed by Black immigrants from the West Indies, and Black immigrants from Europe having worst health outcomes [9].

Two arguments stand out among the various explanations for Black immigrants’ health advantage. First, the immigrant selectivity thesis argues immigrants tend to have characteristics that are positively associated with health. Immigrants tend to be younger, more educated, have a higher level of occupational skill, and are more resilient than their counterparts who did not migrate [31, 32]. Earlier studies suggest immigrant selectivity contributes to health advantages by emphasizing that immigrants of higher socioeconomic status tend to be positively selected on their health status, thus resulting in better health outcomes compared to U.S.-born Black Americans [5, 9, 33, 34]. However, immigrants’ selectivity may not solely be the result of individual characteristics, since structural factors like stringent immigration policies in the U.S. and the socio-political context in their country of origin may be largely responsible for the outcome [35]. The second explanation for Black immigrant’s health advantage is the racial context of origin hypothesis, which argues Black immigrants from predominantly Black and racial mixed countries of origin have a health advantage resulting from a reduced exposure to racism over the life course [5].

## Racial Context of Origin Thesis

Literature examining Black immigrants’ health advantage often focuses on health behaviors as a mechanism to explain the health advantage Black immigrants hold. However, racism is a fundamental cause of social inequalities in health [3, 4]. Thus, we would expect that Black individuals living in contexts where racism is more structurally embedded would have poorer health. Read and Emerson’s Racial Context Hypothesis [5] argues that because of lower exposures to racism, Black immigrants from racially mixed and majority-Black regions have a health advantage over Black immigrants from majority-White contexts [5]. The health status of Black immigrants varies according to their cumulative exposure to racism, where Black individuals living in majority-White environments have been more exposed to structural racism than individuals living in majority-Black or racially mixed environments [5]. This differential exposure should thus create an observable gradient in Black immigrant health where Black immigrants from Africa, South America, and the Caribbean have better health than Black immigrants from Europe and U.S.-born Black Americans. Previous research finds robust to moderate evidence of the Racial Context Hypothesis in terms of self-rated health [8], psychological distress [6], activity limitations and limitations due to hypertension [5], and a host of health behaviors [7]. For example, Hamilton and Hummer [8] found that overall, Black immigrants were less likely to self-report poor/fair health compared to U.S.-born Black Americans with moderate differences found among Black immigrants by country of origin. Immigrants from majority-Black regions such as sub-Saharan Africa reported better health compared to Black immigrants from other regions. This study provides additional evidence to support the racial context of origin hypothesis.

Although Black immigrants arrive in the U.S. in better health than their native counterparts and have a health status comparable to that of U.S.-born White Americans, after a prolonged stay, their health status begins to converge with that of U.S.-born Black Americans [8, 17]. Several factors have contributed to this health deterioration, including the racially tense and discriminatory environment of the U.S. [5, 8]. A longer stay in the U.S. would lead to a worse health for immigrants of color [17]. Although recent Black immigrants may perceive less racial discrimination than U.S.-born Black Americans or Black immigrants who have lived in the U.S. longer [5], their health status continues to be influenced by structural racism the longer they reside in the U.S.. Racial disparities in socioeconomic status are fundamentally caused by racism, and racism itself can have a direct impact on health inequalities through structural and systemic factors. Among African immigrants, one study [18] examined the relationship between discrimination and cardiovascular health risk. The risk of cardiovascular disease among African immigrants who reported frequent experiences of racial and ethnic discrimination was 1.82 times higher than that for African immigrants who reported less frequent experiences. Immigrants from racial and ethnic minority groups may have unique experiences as a result of their racial identity and immigration status, which are further influenced by an array of factors such as inadequate access to resources, residential characteristics, employment opportunities, and inadequate health care [5, 36, 37].

## Data and Methods

### Data

We utilized the 2000 through 2018 waves of Integrated Public Use Microdata Series (IPUMS) Health Surveys, a harmonized version of the National Health Interview Survey (NHIS) [38]. The Integrated Health Interview Series (IHIS) version of the NHIS provides consistency across survey years and allows for the pooling of 19 years of data to increase the sample size of Black immigrant groups. Our subsample was limited to adults 18 years of age or older who self-identified as Black and selected a distinct global region of birth. Our subsample included U.S.-born Black Americans (*N* = 189,126), Black European immigrants (*N* = 743), Black Mexican, Central American, and Caribbean immigrants (*N* = 13,990), Black South American immigrants (*N* = 1320), and Black African immigrants (*N* = 7090).

We categorized U.S.-born Black Americans and Black European immigrants as respondents from majority-White contexts, Black immigrants from Mexico, Central America, the Caribbean, and South America as respondents from racially mixed contexts, and Black immigrants from Africa as respondents from majority-Black contexts. Global region of birth itself does not determine the health outcomes of Black Americans and Black immigrants. Instead, we conceptualized these categories as “markers” to identify individuals in various racial contexts, which may determine levels of exposure to racism [5, 39].

Our analytic sample is on average 43 years of age (SD = 21.48). More than half of the respondents are women (55.14%) and 42% report being married. The majority of the sample has a high school degree or higher (81.44%) with 18.3% reporting a college degree and 32.59% reporting some college. Finally, the majority of the sample (60.52%) report being employed.

### Measures

Our dependent variables included self-rated health, activity limitation, functional activity limitation, lifetime hypertension, and lifetime cancer diagnoses. *Self-Rated Health* is widely used in population and epidemiological studies as a measure of subjective health ratings globally. The measure is typically classified into five categories: poor, fair, good, very good, and excellent. Following previous studies, we dichotomized self-rated health into poor or fair versus good, very good, and excellent health status [40]. As a final measure of self-rated health, we coded 0 for excellent, very good, and good health, and 1 for fair and poor health. *Any Activity Limitation* indicated whether a person had any activity limitation, and *Functional Activity Limitation* measured whether the person had difficulty doing specific activities because of any physical, mental, or emotional problem or illness. Finally, *Lifetime Hypertension* and *Lifetime Cancer Diagnosis* measured whether respondents reported any lifetime diagnosis of hypertension or cancer.

Our main explanatory variable was *Racial Context by Region of Birth*, as described in the subsample above. We utilized U.S.-born Black Americans as the reference group in each model. We also controlled for *Length of Residency* (less than 5 years, 5 to 14 years, and 15 years), as previous research indicates that immigrant health advantages decline with increased duration in the U.S. [5, 8, 41–43]. Following the methodology used in several studies on immigrant arrival and health, we set U.S.-born Black Americans as zero on both the racial context by region of birth variable and the length of residency variable [8, 44–46].

Covariates included *Gender* (coded 0 for men and 1 for women), *Age* (continuous), and *Marital Status* (coded 0 for not married and 1 for married). Socioeconomic status covariates include *Educational Attainment* (less than high school, high school diploma or equivalent, some college, and bachelor’s degree or higher), and *Employment Status* (coded 0 as not employed and 1 for employed).

### Analysis

We first used descriptive statistics to characterize the study sample and estimate the prevalence of each health outcome by region of birth. As our five dependent outcomes are dichotomous variables, we next estimated zero-order logistic regression models for each measure of health. We estimate zero-ordered logistic regression models because they allow us to estimate the direct relationship between racial context of origin and our five physical health outcomes before adjusting for additional covariates. This approach is suitable for our research objective as it allows us to identify the baseline association between racial context of origin and health before considering potentially confounding factors in subsequent models. In terms of our modeling approach, we first tested the relationship between region of birth and each health outcome. Next, we added sociodemographic controls, and finally, we added socioeconomic status controls. We conducted the data analysis using Stata 17 [47] and accounted for the complex survey design of IPUMS Health Surveys by utilizing Stata survey “svy” commands with our models.

## Results

### Descriptive Statistics

Table 1 reports weighted descriptive statistics for respondents across the dependent and independent variables. The proportion of respondents who rated their health as poor or fair was the highest for U.S.-born Black Americans (18.84%), followed closely by immigrants from Mexico, Central America, and the Caribbean, who reported 13.82%. African immigrants and European immigrants reported the lowest fair or poor self-rated health, 5.95% and 6.22%, respectively. The highest percentage of activity limitations was reported by U.S.-born Black Americans (19.70%), while the lowest percentage was reported by African-born Black immigrants (4.56%). Similarly, the distribution of functional limitations in the sample indicated that U.S.-born Black Americans reported the highest levels of functional limitations (34.52%), followed by immigrants from Mexico, Central America, and the Caribbean (24.37%), and immigrants from Africa had the lowest levels of functional limitations (15.61%). Lifetime hypertension was more prevalent among U.S.-born Black Americans (36.64%) than among all groups of Black immigrants in the sample. Black Mexican, Central American, and Caribbean immigrants had the next highest proportion (28.31%), and Black African immigrants had the lowest (17.48%). The lifetime cancer rate among all Black groups, except for Black European immigrants, was generally low. Black European immigrants had the highest lifetime cancer rates, 13.22%, while Black immigrants from South America and Africa had the lowest, 1.32% and 1.41%, respectively.

**Table 1:** Weighted descriptive statistics of sample adult respondents by racial context by region of birth across independent and control variables (*N* = 212,269), National Health Interview Survey 2000–2018

		Region of birth of Black immigrants			
	U.S.-born Black American (*N* = 189,126)	Black European (*N* = 743)	Black Mexican, Central American, and Caribbean (*N* = 13,990)	Black South American (*N* = 1320)	Black African (*N* = 7090)
Poor/fair self-rated health	18.84%	6.22%	13.82%	9.56%	5.95%
Activity limitation					
Any limitation	19.70%	8.06%	9.89%	5.93%	4.56%
Functional limitation	34.52%	21.90%	24.37%	17.18%	15.61%
Hypertension (lifetime)	36.64%	20.86%	28.31%	25.51%	17.48%
Cancer (lifetime)	4.23%	13.22%	3.16%	1.32%	1.41%
Years spent in United States					
Less than 5 years	-	4.73%	8.26%	11.83%	21.79%
5 to 14 years	-	8.94%	24.79%	27.44%	44.17%
15 years or more	-	86.33%	66.95%	60.73%	34.04%
Age	37.3	33.0	44.3	44.7	37.3
Women	55.78%	48.09%	54.78%	47.99%	44.67%
Married	40.37%	43.27%	54.94%	53.98%	58.30%
Educational attainment					
Less than high school	18.81%	4.18%	22.12%	11.03%	10.10%
High school	31.18%	23.34%	29.92%	33.34%	18.57%
Some college	32.94%	39.50%	28.41%	31.73%	32.09%
Bachelor’s degree and higher	17.06%	32.97%	19.55%	23.91%	39.24%
Employed	59.01%	73.14%	68.64%	72.45%	73.75%

Most Black Mexican, Central American, and Caribbean immigrants (66.95%); Black South American (60.73%); and Black European immigrants (86.33%) have resided in the U.S. for 15 years or more. However, most Black African immigrants have lived in the U.S. for shorter periods, with 44.17% living in the U.S. for a period of 5 to 14 years, 21.79% for less than 5 years, and 34.04% for a period of 15 years or more. Black South American immigrants, who averaged 45 years old, were the oldest group in the sample, while Black immigrants from Africa and U.S.-born Black Americans were the youngest (about 37). Women make up a roughly equal percentage of each group, ranging from 44.67% among Black African immigrants to 55.78% among U.S.-born Black Americans. More than half of Black immigrants from Mexico, Central America, the Caribbean, South America, and Africa were married compared with 40.37% of U.S.-born Black Americans and 43.27% of Black European immigrants. The proportion of individuals with a bachelor’s degree among all immigrant groups was higher than that among U.S.-born Black Americans with 17.06% compared to 39.24% among Black African immigrants, 32.97% among Black European immigrants, and 23.91% among Black South American immigrants. Lastly, Black African immigrants and Black European immigrants have similarly high employment rates, with 73.75% and 73.14%, respectively. The rate of employment among U.S.-born Black Americans is the lowest at 59.01%.

### Self-Rated Health

Table 2 displays results from logistic regression models for fair or poor self-rated health, expressed as odds ratios. Model 1 included only self-rated health by region of birth with U.S.-born Black Americans as the reference group. Sociodemographic covariates, including length of residency in the U.S, age, gender, and marital status were added in model 2. In model 3, we introduced socioeconomic status covariates including educational attainment and employment status. In model 1, all Black immigrant groups were significantly less likely to rate their health status as fair or poor compared to U.S.-born Black Americans. In model 2, after adjusting for sociodemographic covariates, all Black immigrant groups were still significantly less likely to report poor or fair health status compared to U.S.-born Blacks Americans. Specifically, in this model, length of residency in the U.S. did not significantly predict self-rated health as poor or fair. Compared to men, women were significantly more likely to rate their health status as poor or fair, and being married reduced the likelihood of rating health status as poor or fair. Health status ratings were also more likely to be considered poor or fair with increasing age. The last model, which included socioeconomic covariates, showed that all Black immigrant groups were less likely to report poor or fair health status compared to U.S.-born Black Americans except for Black European immigrants (adjusted odds ratio, aOR 0.580, *p* = 0.177). Length of residency, age, gender, and marital status were all significant covariates. Having lived in the U.S. for less than 15 years reduced the odds of having poor or fair self-rated health. Health status was more likely to be reported as fair or poor among individuals who were older, women had higher odds than men, and being married reduced the likelihood of rating a person’s health status as fair or poor. When compared to individuals with a bachelor’s degree or higher, people with less education were more likely to rate their health status as poor or fair, and those who were employed were less likely to rate their health status as poor or fair.

**Table 2:** Logistic regression models of fair or poor self-rated health expression in odds ratios (OR)

Region of Birth (Ref. U.S.-born Black)			
Black European	0.286*** (0.0872)	0.451** (0.1350)	0.580 (0.1771)
Black Mexican, Central American, and Caribbean	0.691*** (0.0356)	0.678*** (0.0385)	0.786*** (0.0484)
Black South American	0.456*** (0.0911)	0.450*** (0.0937)	0.616* (0.1333)
Black African	0.273*** (0.0352)	0.401*** (0.0569)	0.611*** (0.0895)
Years spent in the U.S. (Ref. 15 or more years)			
Less than 5 years		0.820 (0.1537)	0.523*** (0.1020)
5 to 14 years		0.922 (0.1138)	0.778* (0.0995)
Age		1.045*** (0.0007)	1.031*** (0.0007)
Women		1.126*** (0.0282)	1.150*** (0.0299)
Married		0.744*** (0.0190)	0.944* (0.0251)
Educational attainment (Ref. Bachelor’s degree +)			
Less than high school			3.381*** (0.1638)
High school			2.354*** (0.1081)
Some college			1.876*** (0.0855)
Employed			0.303*** (0.0088)
Constant	0.232*** (0.0034)	0.031*** (0.0013)	0.042*** (0.0026)

Standard errors in parentheses

Data source: 2000–2018 National Health Interview Survey Adult Sample

*N* = 212,228; ***p < 0.001, **p < 0.01, *p < 0.05

### Activity Limitation

Table 3 displays the results of logistic regression models for any activity limitation and functional activity limitation, expressed as odds ratios. As seen in baseline models, all Black immigrant groups had lower odds of activity limitations compared to U.S.-born Black Americans. Even after adjusting for sociodemographic factors in our second model, we observed the same pattern where all Black immigrant groups reported lower odds of activity limitations than their U.S.-born counterparts. However, for Black Europeans, this relationship was not significant (OR 0.626, *P* = 0.178). In baseline models for functional activity limitation, we observe similar results: all immigrant groups had lower odds of reporting functional activity limitation compared to U.S.-born Black Americans. In final models, Black European immigrants were not significantly different from U.S.-born Black Americans, and all other Black immigrant groups had lower odds of reporting functional limitation. ***p < 0.001, **p < 0.01, *p < 0.05

**Table 3:** Logistic regression models of any and functional limitation expressed in odds ratios (OR)

	Any activity limitation *N* = 212,192			Functional activity limitation *N* = 80,184		
Region of birth (*Ref. U.S.-born Black)*						
Black European	0.357*** (0.1020)	0.626 (0.1776)	0.817 (0.2344)	0.532*** (0.0956)	0.889 (0.1571)	1.044 (0.1833)
Black Mexican, Central American, and Caribbean	0.447*** (0.0232)	0.456*** (0.0264)	0.537*** (0.0345)	0.611*** (0.0282)	0.557*** (0.0302)	0.619*** (0.0352)
Black South American	0.257*** (0.0531)	0.265*** (0.0577)	0.3697*** (0.0854)	0.394*** (0.0549)	0.356*** (0.0503)	0.425*** (0.0616)
Black African	0.195*** (0.0222)	0.350*** (0.0412)	0.556*** (0.0744)	0.351*** (0.0262)	0.532*** (0.0490)	0.678*** (0.0655)
Years spent in the U.S. (*Ref. 15 or more years)*						
Less than 5 years		0.671* (0.121)	0.380*** (0.0748)		0.801 (0.1021)	0.591*** (0.0784)
5 to 14 years		0.662*** (0.0846)	0.560*** (0.0752)		0.874 (0.0811)	0.788* (0.0760)
Age		1.053*** (0.0008)	1.035*** (0.0008)		1.055*** (0.0008)	1.047*** (0.0008)
Women		0.941* (0.0256)	0.931* (0.0266)		1.668*** (0.0393)	1.698*** (0.0409)
Married		0.496*** (0.0133)	0.639*** (0.0180)		0.775*** (0.0184)	0.915*** (0.0222)
Educational attainment (*Ref. Bachelor’s degree* +*)*						
Less than high school			2.401*** (0.1117)			1.655*** (0.0639)
High school			1.822*** (0.0793)			1.370*** (0.0468)
Some college			1.692*** (0.0775)			1.414*** (0.0507)
Employed			0.127*** (0.0043)			0.366*** (0.0088)
Constant	0.245*** (0.0040)	0.029*** (0.0014)	0.078*** (0.0048)	0.527*** (0.0065)	0.038*** (0.0017)	0.065*** (0.0038)

Standard errors in parentheses

Data source: 2000–2018 National Health Interview Survey Adult Sample

***p < 0.001, **p < 0.01, *p < 0.05

### Lifetime Hypertension and Cancer

Table 4 displays the results of logistic regression models for lifetime hypertension and cancer diagnoses, expressed as odds ratios. The odds of lifetime hypertension for all Black immigrant groups were lower than those for U.S.-born Black Americans in baseline models. In model 2, all Black immigrant groups continued to have lower odds of lifetime hypertension than their U.S.-born counterparts after adjusting for sociodemographic factors. For Black Europeans, however, this relationship was not significant (aOR 0.837, *P* = 0.149). The odds of lifetime hypertension were higher for women than for men, and older age was associated with higher odds of having lifetime hypertension. We observed the same trend in the final models as in model 2. Black immigrants in general had lower odds of lifetime hypertension compared to U.S.-born Black Americans. Black immigrants from Europe remained nonsignificant (aOR 0.902, *P* = 0.161). The odds of lifetime hypertension were lower for individuals living in the U.S. for a shorter period as compared to those who have lived in the country for 15 + years. Being older increased the odds of lifetime hypertension, women had higher odds of lifetime hypertension compared to men and being married increased the odds of lifetime hypertension. Among socioeconomic status covariates, a higher level of education is associated with lower odds of lifetime hypertension, with those who have less than a high school education having the highest odds, and those who are employed having lower odds than the unemployed.

**Table 4:** Logistic regression models of lifetime hypertension and cancer expressed in odds ratios (OR)

		Hypertension *N* = 212,183			Cancer *N* = 212,204		
Region of birth (*Ref. U.S.-born Black)*							
Black European		0.456*** (0.0834)	0.837 (0.1486)	0.902 (0.1611)	0.755 (0.3346)	1.704 (0.765)	1.683 (0.7595)
Black Mexican, Central American, and Caribbean		0.683*** (0.0321)	0.572*** (0.0326)	0.596*** (0.0347)	0.739** (0.081)	0.735* (0.0909)	0.771* (0.0947)
Black South American		0.592*** (0.0721)	0.502*** (0.0647)	0.543*** (0.0709)	0.302** (0.1169)	0.313** (0.1213)	0.326** (0.1252)
Black African		0.366*** (0.0247)	0.548*** (0.0468)	0.618*** (0.0534)	0.325*** (0.0644)	0.634* (0.1292)	0.641* (0.1332)
Years spent in the U.S. (*Ref. 15 or more years)*							
Less than 5 years			0.690* (0.1063)	0.622** (0.0975)		0.288** (0.1348)	0.279** (0.1304)
5 to 14 years			0.854* (0.0650)	0.818** (0.0633)		0.834 (0.1820)	0.849 (0.1866)
Age			1.053*** (0.0009)	1.072*** (0.0009)		1.064*** (0.0014)	1.059*** (0.0016)
Women			1.230*** (0.0278)	1.231*** (0.0280)		0.999 (0.0474)	0.976 (0.0461)
Married			1.038 (0.0226)	1.103*** (0.0239)		1.142** (0.0546)	1.164** (0.0567)
Educational attainment (*Ref. Bachelor’s degree* +*)*							
Less than high school				1.352*** (0.0496)			0.742*** (0.0556)
High school				1.270*** (0.0446)			0.797*** (0.0545)
Some college				1.251*** (0.0418)			1.015 (0.0704)
Employed				0.737*** (0.0181)			0.614*** (0.0322)
Constant		0.578*** (0.0077)	0.019*** (0.0008)	0.021*** (0.0012)	0.044*** (0.0010)	0.002*** (0.0001)	0.0031*** (0.0004)

Standard errors in parentheses

Data source: 2000–2018 National Health Interview Survey Adult Sample

*** p < 0.001, **p < 0.01, *p < 0.05

In cancer models, Black Mexican, Central American, and Caribbean immigrants; Black South American immigrants; and Black African immigrants had lower odds of lifetime cancer diagnosis than U.S.-born Black Americans. Black immigrants from Europe were not significantly different from U.S.-born Black Americans (aOR 0.755, *P* = 0.33). In the second model, a similar pattern emerged as in the previous model where Black Mexicans, Central American, and Caribbean immigrants; Black South American immigrants; and Black African immigrants had lower odds of lifetime cancer compared to U.S.-born Black Americans. However, we note that Black Europeans had higher odds of lifetime cancer than U.S.-born Black Americans, although this pattern was not statistically significant. (aOR 1.704, *P* = 0.77). We observed the same trend in our final model as in model 2. Despite recent residency patterns being associated with lower odds of lifetime cancer, the odds were only significant for Black immigrants who have lived in the U.S. for less than 5 years. The odds of lifetime cancer increased with age and with being married. Lastly, having more education and being employed both reduced the risk of cancer over the course of a lifetime.

## Discussion and Conclusion

Black immigrants from Mexico, Central America, the Caribbean, South America, and Africa had better health outcomes when compared to U.S.-born Black Americans. Specifically, we compared different health outcomes between Black immigrants living in the U.S. and U.S.-born Black Americans. Across all health outcomes examined in our study, a consistent finding was that Black immigrants from predominantly Black and racially mixed regions rated their health status as poor or fair less often, experienced less activity or functional activity limitations, and had a lower risk of lifetime hypertension and cancer compared to U.S.-born Black Americans. The significant associations persisted even after controlling for sociodemographic and socioeconomic characteristics. Despite observing a similar pattern of better health outcomes for Black immigrants from Europe compared to U.S.-born Black Americans, these relationships were not significantly different for both groups after controlling for sociodemographic and socioeconomic factors. The only exception was self-rated health status, where Black European immigrants continued to report significantly lower fair or poor self-rated health compared to U.S.-born Black Americans after controlling for sociodemographic variables (age, marital status, length of residency, and gender). The pattern of better health status, however, became nonsignificant when socioeconomic factors were taken into account. This finding suggests first that Black immigrants from majority-White contexts have worse health outcomes than Black immigrants from predominantly Black and racially mixed contexts. In addition, Black immigrants from majority-White contexts tend to have similar health patterns as U.S.-born Black Americans compared to immigrants from other contexts. We present additional evidence in this study to support the Racial Context Hypothesis [5], by establishing that Black immigrants from predominantly Black and racially mixed regions of origin have a health advantage over Black immigrants from predominantly White regions in the case of self-rated health, activity limitation, lifetime hypertension, and cancer incidence. According to the hypothesis, these differences are associated with varying levels of exposure to racism and racial inequality in distinct contexts. Prolonged exposure to racial discrimination results in adverse health effects [3, 4].

Several interrelated factors could explain this pattern. First, the socio-political contexts of the immigrant’s country of origin plays an important role. Immigrants from majority-White contexts may come from countries where racial hierarchies are deeply entrenched and institutionalized. Thus, living in predominantly Black or racially mixed regions is likely associated with lesser exposure to racial discrimination while living in majority-White regions may be associated with greater exposure to systemic and interpersonal racism. Cumulative exposure to racial discrimination can lead to chronic stress with resulting adverse effects on physical health. Second, greater exposure to racial discrimination among Black individuals results in greater predicted risk for unhealthy behaviors and coping mechanisms—all of which are associated with poorer physical health outcomes.

Although there is overwhelming evidence [40, 48, 49] demonstrating that the initial health advantage immigrants have over their native-born counterparts declines with prolonged length of residency in the U.S. [8, 17, 40, 48], we found that length of residency did not significantly predict Black immigrants’ health for self-rated health and functional activity limitation until after controlling for socioeconomic characteristics. Furthermore, where there is some evidence in the literature [31, 32, 50] that argues for the healthy immigrant selectivity thesis where people who migrate are said to have certain positive attributes that make them likely to be successful in their host country (e.g., better educational status that provides the right credentials for better employment prospects and social capital), we found that Black European immigrants do not significantly differ from U.S.-born Black Americans across all health outcomes in our study. Thus, the immigrant health selectivity effect only partly accounts for the health advantage of Black immigrants. Further, although studies [31, 32, 50] suggest immigrants have an advantage in health as a result of their socioeconomic status, our findings indicate that socioeconomic status does not fully explain current patterns for Black immigrants, as we continue to find significant differences in health outcomes by region of birth, even after controlling for sociodemographic and socioeconomic factors except in the case of Black European immigrants. As a result, our study provides substantial evidence for the Racial Context Hypothesis [5] suggesting that there is an association between systems of racial oppression and Black immigrants’ health outcomes.

While our study contributes to understanding the physical health outcomes of Black immigrants, the conclusions should be interpreted in light of the following limitations. There were relatively few Black immigrants from Europe and South America compared to other regions in our sample. This may have contributed to some of the nonsignificant relationships we observed in our models. Additionally, because we used only publicly available NHIS data, we were unable to examine additional characteristics of the region of birth, such as healthcare systems and socioeconomic characteristics of the place of origin, which may influence the health outcomes of Black immigrants. Finally, because our study relies on cross-sectional data from the NHIS, we are unable to specifically identify the causal mechanisms underlying these health outcomes.

Further, there are several avenues for further research. One potential direction is to analyze the longitudinal impact of the racial context of origin on health outcomes by following the health trajectories of Black immigrants from different racial contexts as they continue to live in the U.S. over time. Additionally, research could examine how other intersectional social positions and statuses beyond immigrant status and race could provide a deeper understanding on the health patterns of Black immigrants in the U.S. Another future possible direction is to utilize country-specific data that would allow for a more detailed exploration of how specific social and political contexts of individual countries of origin shape the health status of Black immigrants in the U.S. Lastly, as the racial dynamics and policy environment in both sending and receiving countries continue to evolve, future research could examine how these socio-political changes influence the health patterns of Black immigrants in the U.S. The immediate next phase of our research examines additional health outcomes, specifically heart health outcomes, to examine if a host of heart health outcomes vary by racial context of origin.

Nonetheless, our study contributes in a unique way to the ongoing discussion regarding the health of Black immigrants. Our study extends the test of the Racial Context Hypothesis to additional physical health outcomes and utilizes additional years of data to replicate previous findings. Furthermore, unlike other research on the health of Black immigrants, our analysis focuses on Black immigrant’s region of origin. Thus, by distinguishing Black immigrants by region of origin, our results indicate that Black immigrants are not a homogeneous group with the same health outcomes and social backgrounds, and the “Black immigrant advantage” varies when considering racial context of origin.

## Data Availability

Blewett, Lynn A, Julia A Rivera Drew, Miriam L King, and Kari C W Williams. 2019. “IPUMS Health Surveys: National Health Interview Survey, Version 6.4 [Dataset].” IPUMS. IPUMS. 10.18128/D070.V6.4.
